# Influence of milking units and working vacuum level on the mechanical milking of goats

**DOI:** 10.1111/asj.13667

**Published:** 2021-12-07

**Authors:** Maria Caria, Carlo Boselli, Angela Calvo, Silverio Grande, Giuseppe Todde

**Affiliations:** ^1^ Department of Agricultural Sciences University of Sassari Sassari Italy; ^2^ Istituto Zooprofilattico Sperimentale del Lazio e della Toscana Rome Italy; ^3^ Dipartimento di Scienze Agrarie, Forestali e Alimentari University of Turin Torino Italy; ^4^ Associazione Nazionale della Pastorizia Rome Italy

**Keywords:** goat, liner, milk flow curve, milkability, working vacuum

## Abstract

The objectives of this study were to evaluate and compare the effect of working vacuum levels (35 and 44 kPa) and liners dimensions (mouthpiece lip diameter and overall length, 20–185 and 22–170 mm) on the main milking characteristics of goats. The results highlight that both the working vacuum level and the liner dimension have influenced the milk flow curve parameters. The maximum variations were found for peak flow rate, which increased significantly with liner dimensions of 20–185 mm at a working vacuum level of 44 kPa as well as average milk flow rate and for plateau phase duration. The incorrect adoption of operative parameters and unsuitable milking machine components, might affect the performance of the mechanical milking and negatively affecting animal productions and welfare.

## 1 INTRODUCTION

The milking machine is one of the main innovative technologies that allowed to increase the productivity of work in dairy farms. However, mechanical milking remains capable of generating induced effects that are decisively reflected on animal management (Díaz et al., 2013; Hamann et al., 1994). The fine‐tuning of the milking machine through the settings of the operative parameters allows adapting the mechanical action of the system to the animal species, whereas the correct sizing and choice of the system components contribute to the regular milking process in terms of vacuum stability, milking times, and animal comfort.

The working vacuum level is one of the milking parameters that are regulated at different levels depending on the type of the machine milking and the specific milk emission curve of the bred species. As observed in Italian goat farms, the working vacuum level is generally set between 41 and 44 kPa, and only a small percentage of systems milks at 37–41 kPa (Pazzona & Murgia, 2003), whereas lowest values (35 kPa) were found by Billon et al. (1999).

The technical and scientific knowledge on milking units, with specific reference to the physical properties of the liner design, influence the milking of the different species, as has been shown by specific studies carried out on dairy cows (Mein et al., 1983; Zucali et al., 2009) and on dairy sheep and goats (Alejandro et al., 2014; Le Du, 1982, 1985).

The liners available on the market have different shapes and dimensions for the same dairy species, in accordance with the morphological differences of the mammary gland (Strapák et al., 2018; Tančin et al., 2006). Milking liners with a narrow opening can generate a compression at the base of the teat. On the contrary, a wide opening can cause the liner climbing in high working vacuum settings. The liner length also affects the number of intramammary infections and compromises the benefits of the pulsation on the teat (Mein et al., 1983).

On the whole, incorrect management techniques constitute important stressful causes for animals, producing negative effects on the health status, on the quality of the productions and on their milkability (Labussière, 1984; Zucali et al., 2019).

To investigate this problem, the present study aims to highlight the results of comparative tests carried out to evaluate the effect of the working vacuum levels and the dimensions of the liners on the main milking characteristics of the goats.

## 2 MATERIALS AND METHODS

The study was carried out on a goat dairy farm located in Italy over a period of 8 days. A total of 42 Alpine goats of different parity (2.86 ± 1.34) and at different Days In Milking (DIM: 80.00 ± 13.23) were milked twice a day in 20 stalls milking parlor equipped with clusters Almatic™ (Delaval, Tumba, Sweden). During the trials, two working vacuum levels (35 and 44 kPa) and two different type of liners, which were distinguished by the mouthpiece lip diameter and overall length (20–185 and 22–170 mm), were tested. The liners were both conical, made of synthetic rubber and specifically produced for goats by Delaval (Tumba, Sweden). The milking machine was set at a 90 cycles/min pulsator rate and a 50% pulsator ratio. Milk flow curves were recorded and milk samples were collected after milking from all of the animals in lactation on different combinations of working vacuum and liner dimensions. The treatments designed were: 44 kPa and 20–185 mm; 44 kPa and 22–170 mm; 35 kPa and 22–170 mm; 35 kPa and 20–185 mm. Specifically, the 42 goats received the same treatment per two consecutive days (four milkings). Among the 672 recorded milk flow curves, 12 (1.8%) were discarded as they did not achieve the minimum necessary flow to return the main parameters. Thus, at the end of the test, a total of 660 flow curves were analyzed, and as many qualitative‐quantitative milk samples, to evaluate the effect of four different combinations of working vacuum and liner dimensions on milk fractioning, milk flow, milking time and milk quality.

### 2.1 Milk flow curves

The milk flow curves were recorded during the morning and evening milking sessions using two electronic mobile milk flow meters (LactoCorder®; WMB, Balgach, Switzerland), as used in previous studies with goats measurement program (Bašić et al., 2009; Caria et al., 2013; Šlyžiene et al., 2016; Zucali et al., 2019). Variables measured per milking were the following:

Milk yield (MY, kg): total milk yield per head per milking from the beginning to the end of the mechanical milking; 1MY (kg): milk yield from the beginning of measurement to completion of 1 min measuring time; 2MY (kg): milk yield from the beginning of measurement to completion of 2 min measuring time; 3MY (kg): milk yield from the beginning of measurement to completion of 3 min measuring time; Effective Milking Time (EMT, min): between attaching the teat cup and reaching the value of 0.20 kg/min at the end of milking; Peak Flow Time (PFT, min): between attaching the teat cup and reaching the peak flow; Milk Ejection Time (MET, min): from milk flow rate >0.25 kg/min until milk flow decreased below 0.20 kg/min; Time of Plateau Phase (PPT, min): the time of phase with a high constant milk flow, from the vertex of the incline phase to the vertex of the decline phase; Lag Time (LT, min): from the beginning of measurement until a 0.25 kg/min threshold in the milk flow was reached; Peak Flow Rate (PFR kg/min) in the main milking process within a time interval of eight measuring points (22.4 s); Average milk Flow Rate (AFR, kg/min): during milk ejection time.

### 2.2 Milk samples

At each milking a milk sample representative of the whole milking was automatically collected by Lactocorder for each animal to be analyzed for milk quality parameters. After collection, milk samples were refrigerated (5°C) and transported to the milk laboratory of Istituto Zooprofilattico Sperimentale del Lazio e Toscana (Rome, Italy). Content of fat (%), protein (%), lactose (%), casein (%), and milk urea nitrogen (MUN) (mg/dl) were detected by MilkoScan™ FT6000 (FOSS Electric, Hillerød, Denmark) using FTIR spectrophotometry calibrated with appropriate goat standards. Somatic cell count (SCC) (n/ml) were detected by Fossomatic FC (FOSS Electric, Hillerød, Denmark) using a fluoro‐optometric method. To achieve a normal distribution, a logarithmic transformation (log10) was applied to SCC (Ali & Shook, 1980).

### 2.3 Statistical analysis

To evaluate the effect of the working vacuum levels, liner dimensions, and their interaction on the main milking characteristics of the goats, the data were analyzed by the PROC MIXED procedure of SAS version 9.2 (SAS Institute Inc., Cary, NC, USA), with the following mixed linear model:

$$\text{Yijkl}=\mathrm{Vi}+\mathrm{Dj}+\left(V\times D\right)k+\mathrm{Al}+\text{Eijkl}$$

where

Vi = fixed effect of the working vacuum (35, 42 kPa),

Dj = fixed effect of liner dimensions (20–185, 22–170 mm),

(V × D)k = fixed effect of the working vacuum × diameter interaction,

Al = random effect of animal,

Eijkl = random residual.

## 3 RESULTS

In general, both the working vacuum level and the liner dimensions have influenced, though to different degrees, the milk flow curve parameters (Table 1).

**TABLE 1:** Effect of vacuum level (kPa) and liner dimension (mm) on milk and milking parameters (LS‐mean ± standard error)

Variables	Vacuum (kPa)			Liner dimensions (mm)		
	35	44	*p* value	20–185	22–170	*p* value
MY (kg)	1.24 ± 0.027^a^	1.05 ± 0.027^b^	0.000	1.15 ± 0.027^a^	1.14 ± 0.027^a^	0.720
1MY (kg)	0.67 ± 0.017^b^	0.73 ± 0.017^a^	0.000	0.73 ± 0.017^a^	0.67 ± 0.017^b^	0.002
2MY (kg)	1.05 ± 0.022^a^	0.94 ± 0.022^b^	0.000	1.02 ± 0.022^c^	0.97 ± 0.022^d^	0.049
3MY (kg)	1.16 ± 0.024^a^	0.98 ± 0.024^b^	0.000	1.09 ± 0.024^a^	1.05 ± 0.024^a^	0.155
EMT (min)	2.51 ± 0.059^a^	2.13 ± 0.059^b^	0.000	2.22 ± 0.059^b^	2.42 ± 0.059^a^	0.000
PFT (min)	1.35 ± 0.050^a^	0.90 ± 0.050^b^	0.000	1.04 ± 0.050^b^	1.21 ± 0.050^a^	0.000
MET (min)	1.92 ± 0.058^a^	1.35 ± 0.058^b^	0.000	1.63 ± 0.058^a^	1.64 ± 0.058^a^	0.889
PPT (min)	0.93 ± 0.048^a^	0.40 ± 0.048^b^	0.000	0.52 ± 0.048^b^	0.80 ± 0.048^a^	0.000
LT (min)	0.37 ± 0.018^a^	0.36 ± 0.018^a^	0.628	0.33 ± 0.018^b^	0.39 ± 0.018^a^	0.002
PFR (kg/min)	0.75 ± 0.017^a^	0.77 ± 0.017^a^	0.366	0.79 ± 0.017^a^	0.73 ± 0.017^b^	0.001
AFR (kg/min)	0.61 ± 0.016^b^	0.69 ± 0.016^a^	0.000	0.67 ± 0.016^a^	0.63 ± 0.016^b^	0.015
Casein (%)	2.21 ± 0.026^b^	2.34 ± 0.026^a^	0.000	2.30 ± 0.026^a^	2.25 ± 0.026^b^	0.006
Lactose (%)	4.21 ± 0.017^b^	4.37 ± 0.017^a^	0.000	4.25 ± 0.017^b^	4.33 ± 0.017^a^	0.000
Fat (%)	3.43 ± 0.047^b^	3.62 ± 0.047^a^	0.002	3.46 ± 0.047^b^	3.58 ± 0.047^a^	0.048
Protein (%)	3.01 ± 0.029^b^	3.10 ± 0.029^a^	0.000	3.06 ± 0.029^a^	3.06 ± 0.029^a^	0.953
Urea (mg/dl)	43.90 ± 0.533^b^	45.56 ± 0.534^a^	0.001	43.09 ± 0.532^b^	46.37 ± 0.534^a^	0.000
SCC (Log_10_)	5.36 ± 0.046^a^	5.30 ± 0.046^a^	0.094	5.39 ± 0.046^a^	5.27 ± 0.046^b^	0.000

*Note*: MY: Milk Yield; 1MY: milk yield of first minute; 2MY: milk yield of 2 min; 3MY: milk yield of three; EMT: Effective Milking Time; PFT: Peak Flow Time; MET: Milk Ejection Time; PPT: Time of Plateau Phase; LT: Lag Time; PFR: Peak Flow Rate; AFR: Average milk Flow Rate; SCC: Somatic Cell Count. a–d: Means within a row with different letters are significantly different (*p* < 0.05).

The milk yield, as well as the quantity of milk produced in the first minutes, was greater using the lower vacuum level, except for the milk obtained in the first minute of milking. As expected, the reduction in the working vacuum level led to an increase in almost all the four milking time variables. Instead, the lag time to 0.25 kg did not show significant differences between the two working vacuum levels considered. The variables related to milk flow rates were higher with a working vacuum of 44 kPa, specifically the peak and average flow rate.

In reference to the qualitative analyses performed on the milk samples, the fat content was higher with a working vacuum of 44 kPa (3.62%–3.43%), a similar result was found for the protein content (3.10%–3.01%), lactose (4.37%–4.21%), casein (2.34%–2.21%), and urea (45.56–43.90 mg/dl). The results obtained with the two types of liner diameter sizes also showed major differences for SCC (Log10) and peak flow rate, that increased with the liner lower dimension (20–185 mm) and for lactose, urea, effective milking time, time for maximum flow and plateau phase duration that increased with a liner dimension of 22–170 mm (*p* < 0.001).

A significant interaction between the working vacuum levels and liner dimensions for milk and milking parameters measured has been found (Table 2). A higher (*p* < 0.05) increase in lactose occurred with the liner dimensions of 20–185 mm at a working vacuum level of 44 kPa, compared to measurements performed at 35 kPa.

**TABLE 2:** Interaction of working vacuum level (kPa) and liner dimensions (mm) on milk and milking parameters

Variables	20–185			22–170			Vacuum × diameter *p* value
	35	44	s.e.d.	35	44	s.e.d.	
MY (kg)	1.25	1.05	0.038	1.22	1.05	0.039	0.608
1MY (kg)	0.68	0.77	0.024	0.65	0.69	0.025	0.267
2MY (kg)	1.08	0.96	0.033	1.02	0.93	0.034	0.516
3MY (kg)	1.19	0.99	0.038	1.14	0.97	0.038	0.493
EMT (min)	2.43	2.00	0.080	2.59	2.26	0.081	0.403
PFT (min)	1.26	0.81	0.066	1.44	0.98	0.066	0.940
MET (min)	1.95	1.32	0.073	1.90	1.38	0.073	0.330
PPT (min)	0.83^b^	0.20^d^	0.067	1.02^a^	0.59^c^	0.068	0.037
LT (min)	0.32	0.34	0.026	0.41	0.37	0.026	0.160
PFR (kg/min)	0.77	0.80	0.024	0.73	0.73	0.024	0.378
AFR (kg/min)	0.61^b^	0.73^a^	0.021	0.60^b^	0.66^b^	0.022	0.033
Casein (%)	2.23	2.37	0.022	2.20	2.31	0.02	0.217
Lactose (%)	4.12^c^	4.38^a^	0.020	4.30^b^	4.35^ab^	0.02	0.000
Fat (%)	3.38	3.54	0.087	3.48	3.69	0.09	0.665
Protein (%)	3.01	3.10	0.024	3.02	3.10	0.02	0.899
Urea (mg/dL)	41.00^b^	45.18^a^	0.714	46.80^a^	45.94^a^	0.72	0.000
SCC (Log10)	5.43	5.36	0.046	5.29	5.25	0.05	0.764

*Note*: MY: Milk Yield; 1MY: milk yield of first minute; 2MY: milk yield of 2 min; 3MY: milk yield of three; EMT: Effective Milking Time; PFT: Peak Flow Time; MET: Milk Ejection Time; PPT: Time of Plateau Phase; LT: Lag Time; PFR: Peak Flow Rate; AFR: Average milk Flow Rate; SCC: Somatic Cell Count. a–d: Means within a row with different letters are significantly different (*p* < 0.05).

Regarding milking parameters, the maximum variations were found for peak flow rate, which increased significantly with liner size of 20–185 mm at a vacuum level of 44 kPa as well as average milk flow rate, and for plateau phase duration. No significant interactions between the working vacuum level and liner dimensions for the other parameters have been found. However, milk yield tended to be higher with the vacuum level of 35 kPa, (Table 2).

## 4 DISCUSSION

The study showed that the effective milking time was less than 15% using the highest working vacuum level (44 kPa). The reduction of the effective milking time would determine an increase in terms of throughput of the milking system of about 12 goats/h, simulating a milking routine without pre‐stimulation and using automatic cluster removers (Caria et al., 2011). The results obtained showed that reducing the working vacuum level up to 35 kPa did not significantly change the duration of the lag time (about 20 s), in agreement with the results found for other dairy species (Atigui et al., 2014; Caria et al., 2012). The increase in milking times, related to the main phase and the maximum peak flow, probably, affected the significant growth in the amount of total milk obtained per animal using a working vacuum level of 35 kPa rather than of 44 kPa, as also found by other authors (Caria et al., 2011, 2012; Zucali et al., 2019). The values found for the average milk flow in the main milking phase increased with the increase in the working vacuum by 13.1%, as confirmed by other studies carried out on dairy goats (Zucali et al., 2019) and on other dairy species (Besier & Bruckmaier, 2016).

The milking vacuum level set at 44 kPa compared to 35 kPa allowed to obtain higher values for all the variables related to the composition of the milk, with the exception of the SCC. In particular, the largest increase occurred in Casein + 5.9% and Fat + 5.5% (Lactose + 3.4%, Protein + 3.0%, Urea + 3.8%). The content of fat in milk was significantly affected by the vacuum level as found also by Zucali et al. (2019). The SCCs, on the other hand, showed no difference with the variation of the working vacuum levels, confirming what other studies on small ruminants have found (Fernández et al., 2019; Zucali et al., 2019).

From the values obtained, the liners with a smaller mouthpiece lip diameter/overall length ratio (0.11 vs. 0.13) provided better milking performance in terms of effective milking time. In fact, liners with a smaller mouthpiece lip diameter significantly decrease the milk lag time by 15.4%, keeping the milk ejection time unchanged. Liners with a longer body and narrower mouthpieces provided better  performance in terms of cheese yield (Casein + 2.22%). In particular, a negative aspect was highlighted in the milk with this type of liner (mouthpiece lip diameter/body length ratio 0.11), where the amount of SCC increased significantly.

The results obtained show that the interaction between the liner and the working vacuum is statistically significant only for the plateau phase duration (*p* ≤ 0.05), average milk flow rate (*p* ≤ 0.05), lactose, and urea (*p* ≤ 0.001). Table 2 shows that the effects produced by the 44 kPa and 20–185 mm thesis, compared to the other combinations, allowed to increase the amount of lactose, urea and average milk flow rate (6.3%, 10.2%, and 21.7% respectively), as well as having the lower plateau phase duration (−80.4%).

The working vacuum level significantly affected the 82% of the variables considered (14 out of 17), while the mouthpieces diameter/liner length ratio influenced 77% of the variables (13 out of 17). Liner dimensions (20–185 mm vs. 22–170 mm) significantly influenced the effective milking time but not the milk yield. The working vacuum level at 35 kPa causes a greater quantity of milk in more time, while at 44 kPa determined higher values on the variables concerning the composition of the milk. The combined effect of the two parameters remained limited to 24% (four out of 17) of the variables considered.

Based on the results obtained in this study, the optimal combination of the working vacuum level and liner dimensions was 35 kPa with 20–185 mm, this arrangement allowed to have the highest milk production in the lowest milking time.

It is important to underline that, improper maintenance of the milking machine, specifically an incorrect adoption of liner type and operative parameters, might affect the performance of the mechanical milking such as milking time and milk yield. Thus, the introduction of standardized indicators, specific to each species, will support the application of livestock production techniques more suitable to animal welfare.

## CONFLICT OF INTEREST

All authors declare no conflict of interest.
